# Supplementary figures and images for: Jumping DNA polymerases in bacteriophages
Source: bioRxiv. 2024 Apr 27:2024.04.26.591309. Preprint. [Version 1] doi: 10.1101/2024.04.26.591309 (PMC11188092; doi:10.1101/2024.04.26.591309)

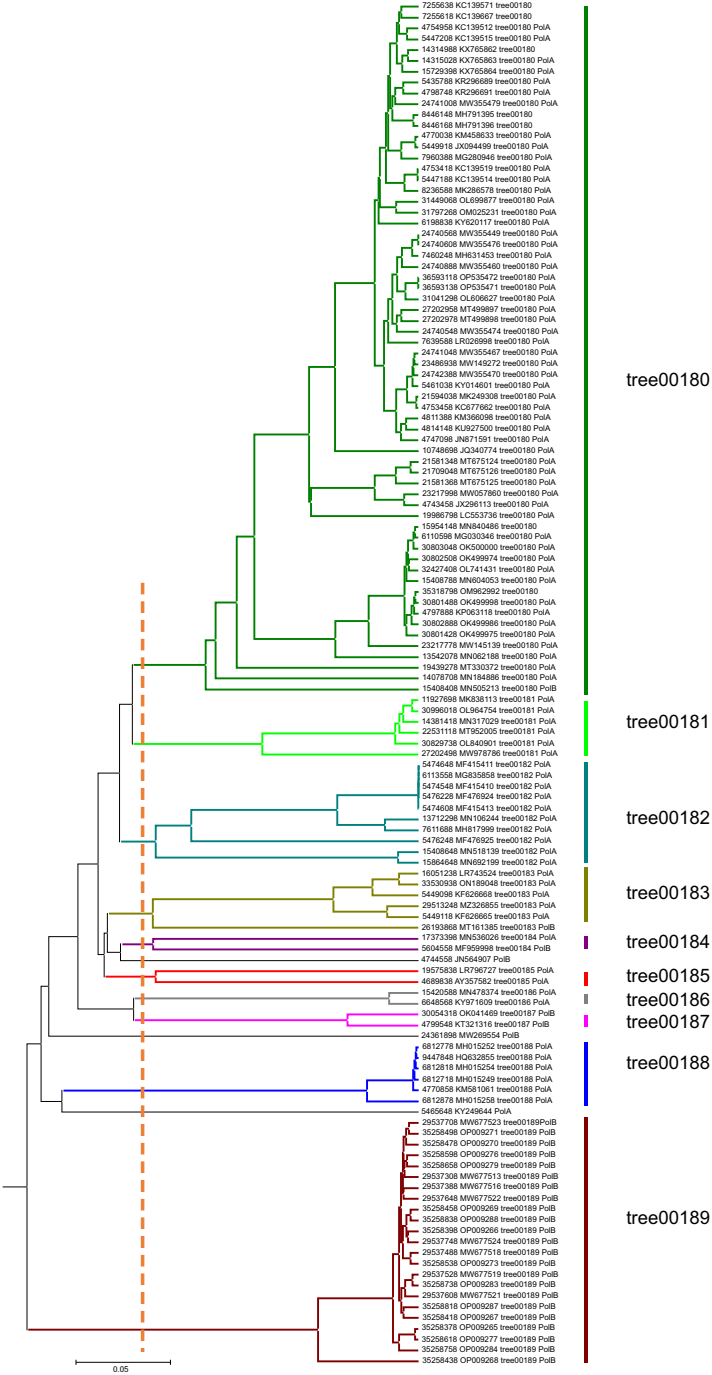

Supplement: Supplement 1 [file media-1.pdf]

PolA

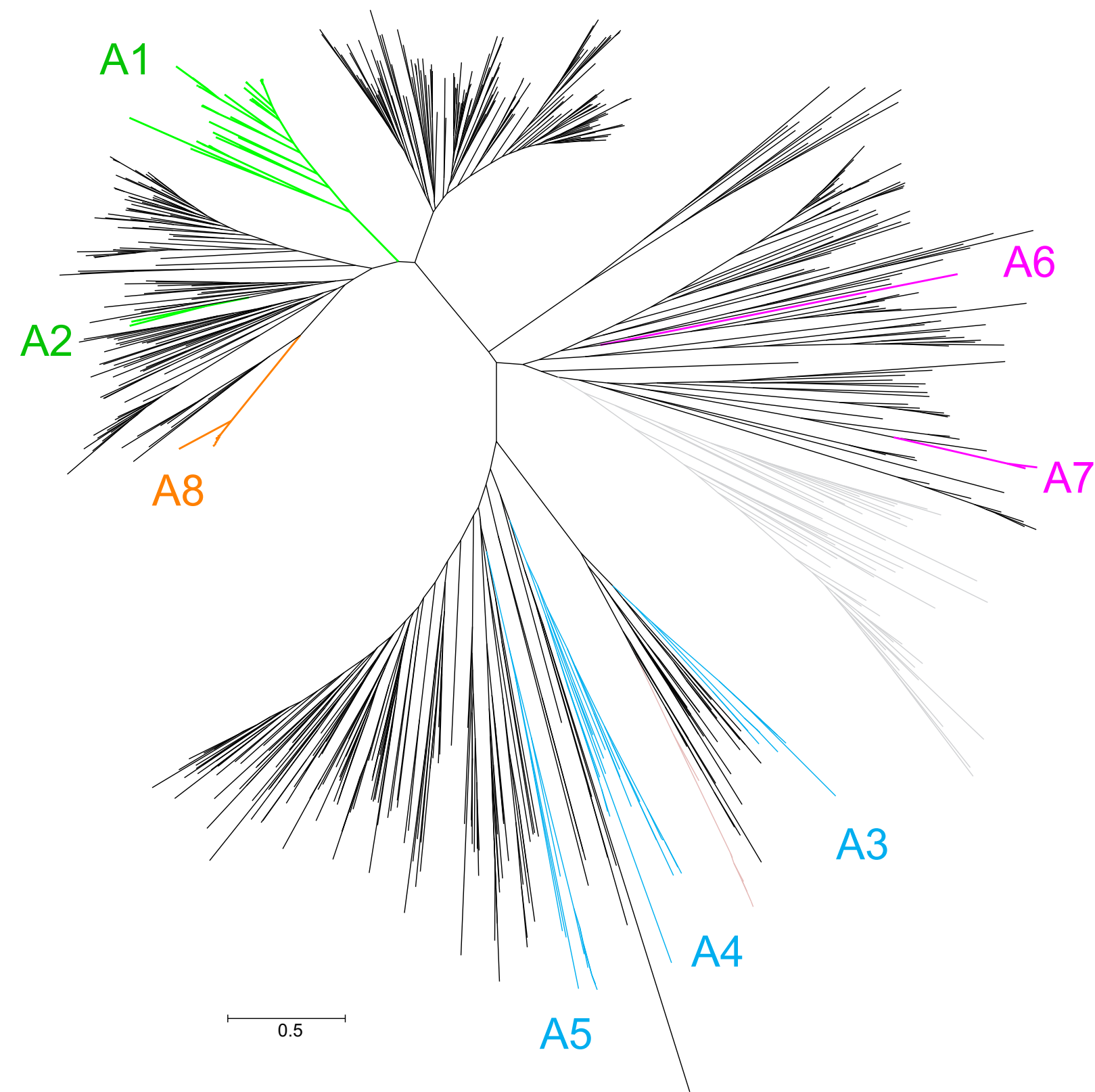

PolB

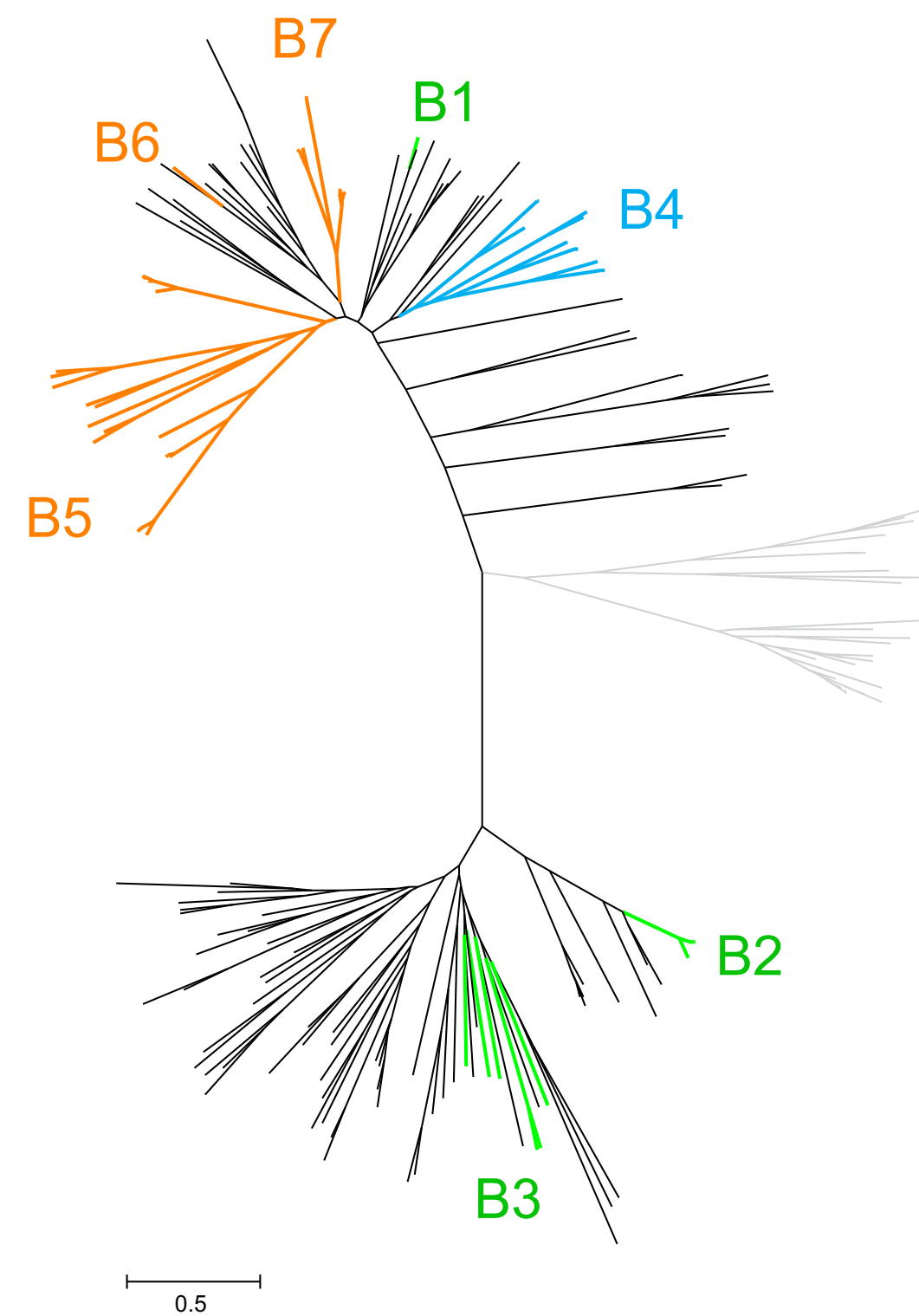

PolC

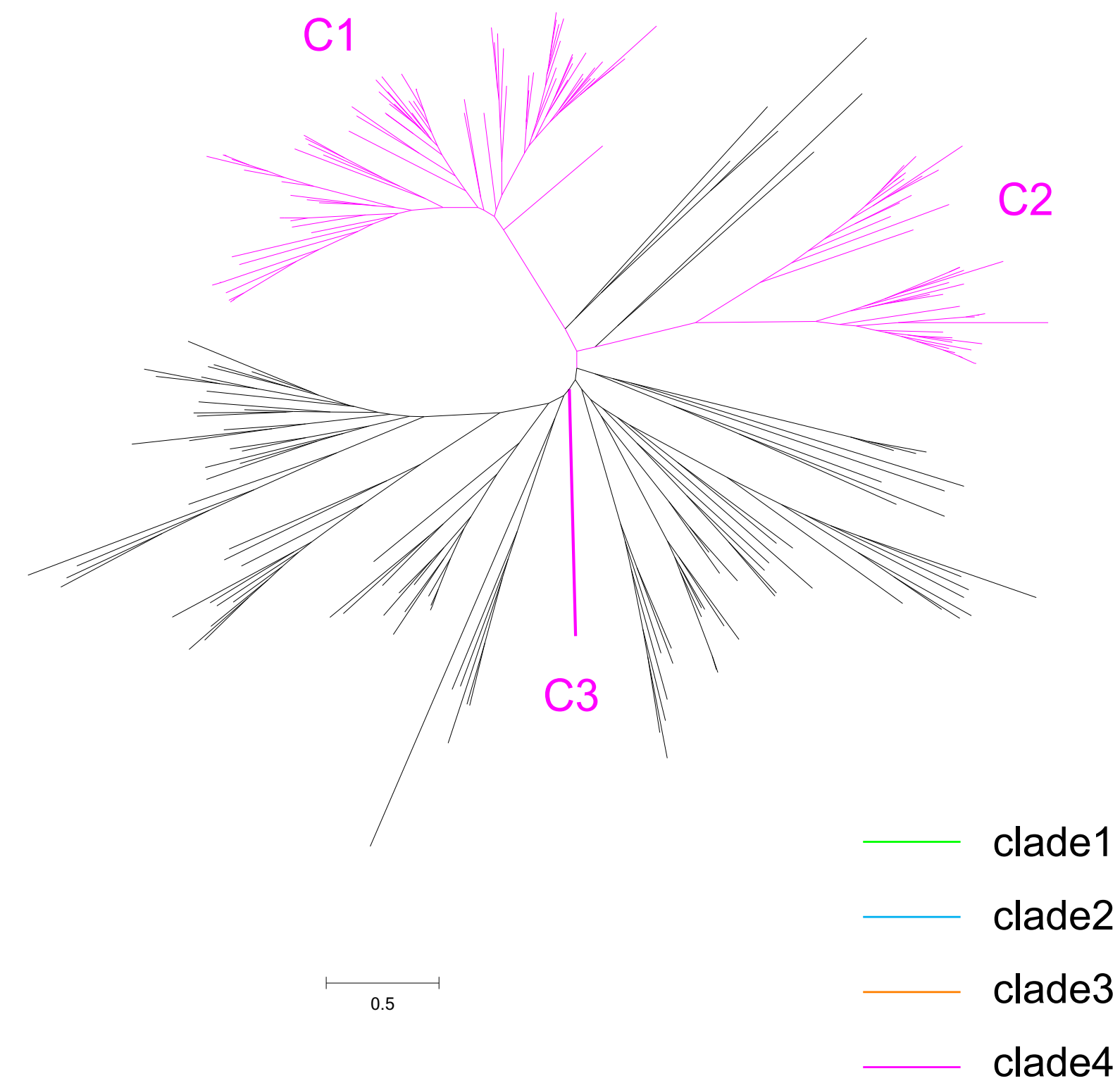

- clade1
- clade2
- clade3
- clade4

Supplement: Supplement 2 [file media-2.pdf]

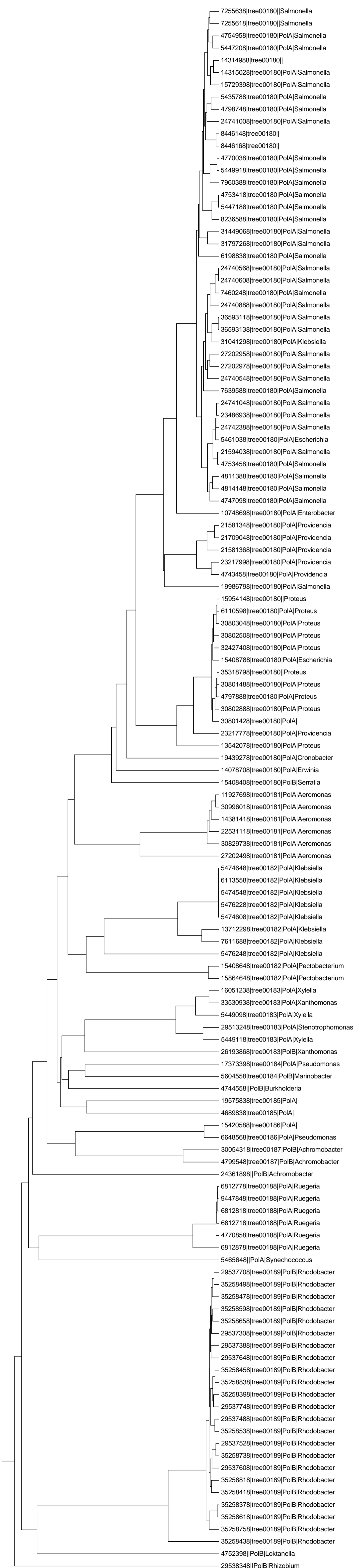

0.050

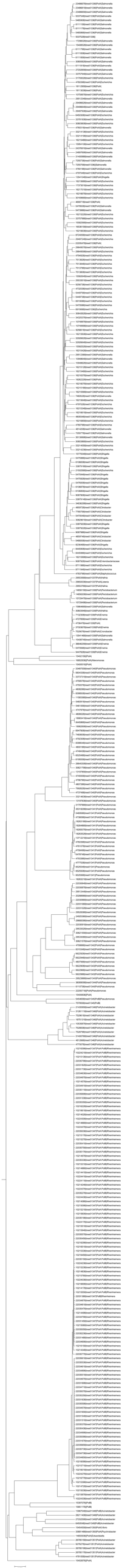

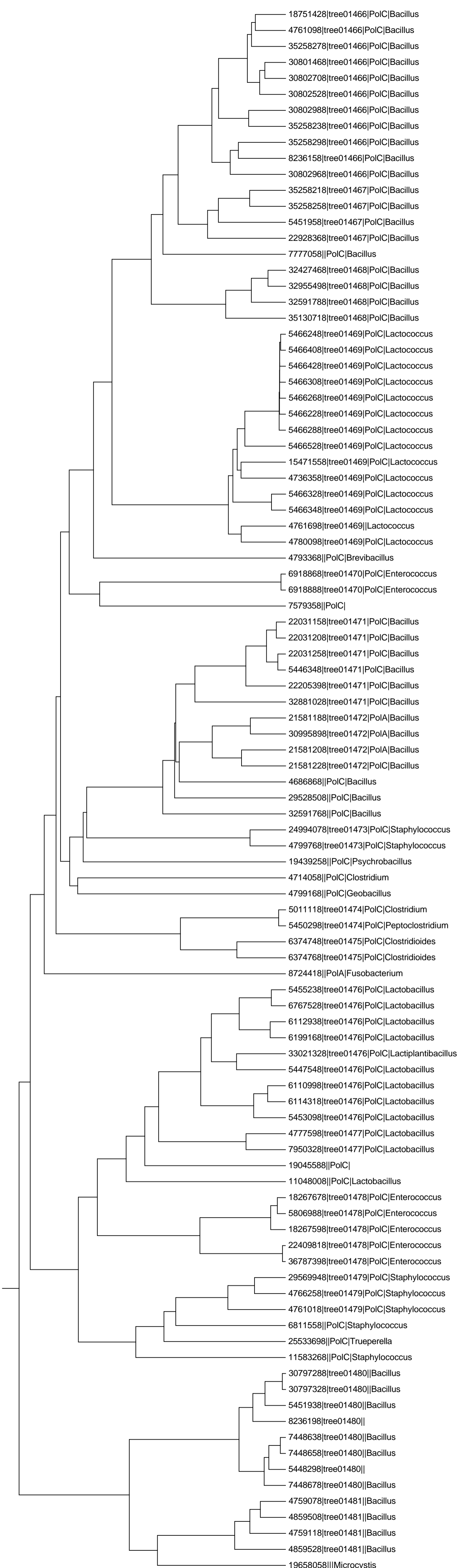

0.10

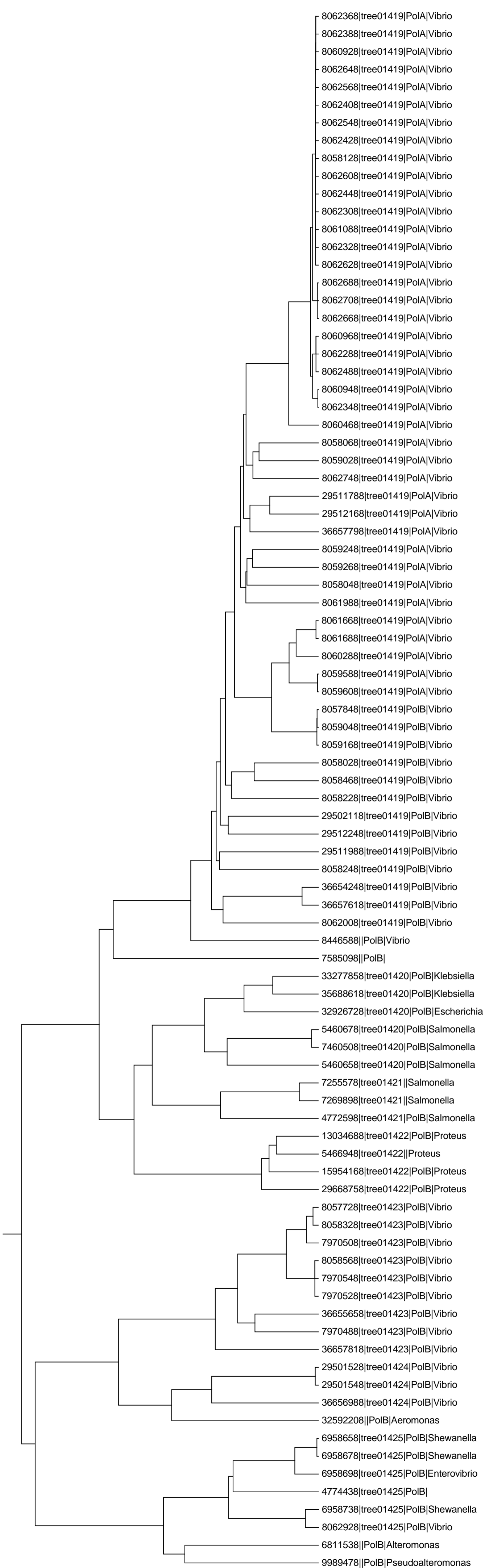

0.050

Supplement: Supplement 3 [file media-3.pdf]
